# The effect of Annexin A5 overexpression on invasiveness and expression of the genes involved in epithelial-mesenchymal transition of HCT 116 cell line

**DOI:** 10.22099/mbrc.2023.47160.1823

**Published:** 2023

**Authors:** Bahareh Zamani, Amin Ramazani, Jamileh Saberzadeh, Puria Rostampour, Mohammad Ali Takhshid

**Affiliations:** 1 Division of Medical Biotechnology, Department of Laboratory Sciences, School of Paramedical Sciences, Shiraz University of Medical Sciences, Shiraz, Iran; 2 Shiraz Institute for Cancer Research, School of Medicine, Shiraz University of Medical Science, Shiraz, Iran; 3 Diagnostic Laboratory Sciences and Technology Research Center, Paramedical School, Shiraz University of Medical Science, Shiraz, Iran

**Keywords:** Colorectal cancer, Epithelial to mesenchymal transition, Metastasis, *ANXA5*

## Abstract

Epithelial-to-mesenchymal transition (EMT) plays a critical role in colorectal cancer (CRC) metastasis. In the present study, we evaluated the effects of annexin A5 (*ANXA5*) overexpression on invasiveness as well as the expression of genes involved in EMT of HCT 116 cell line. PCMV6-AC-IRES-GFP plasmid harboring *ANXA5* cDNA was constructed. HCT 116 cell line was transfected with recombinant plasmids using Lipofectamine 3000. Fluorescent microscopy was used to determine the efficiency of plasmid transfection. Cell viability was determined using the MTT assay. HCT 116 cell migration was evaluated using wound healing assay and transwell migration assay. Real-time quantitative polymerase chain reaction (RT-qPCR) was used to measure the expression of genes involved in EMT. The results of RT-qPCR showed overexpression of *ANXA5* compared to the control group. *ANXA5* overexpression had no significant effects on cell viability but significantly decreased the rate of wound closure in the wound healing assay as well as the number of migrated cells in transwell assay. Furthermore, *ANXA5* overexpression decreased the expression of N-cadherin, Snail, Slug, MMP-2, and MMP-9 while the expression of E-cadherin increased following *ANXA5* overexpression. However, VEGF expression did not significantly change after *ANXA5* overexpression. Results of the present study suggest that *ANXA5* overexpression might have inhibitory effects on the metastasis of CRC through modulating the expression of EMT- related genes.

## INTRODUCTION

Chemotherapy, surgical resection, and radiation therapy are routine methods that are currently used for treating colorectal cancer (CRC). However, metastasis to distant organs decreases the efficacy of these therapies, and CRC remains lethal cancer worldwide [1]. Therefore, understanding molecular mechanisms involved in CRC metastasis is important and has attracted great attention in cancer research. One of the essential mechanisms underlying cancer metastasis is the epithelial-to-mesenchymal transition (EMT), a complex cellular and molecular process that is associated with abnormalities in the structure and function of adhesions molecules, cytoskeleton, and matrix metalloproteinases (MMPs) [2]. At the molecular level, EMT is identified with several characteristics notably decreased E-cadherin and increased N-cadherin expression (cadherin switching), and increased MMPs expression. Increased expression and activity of MMPs degrade the extracellular matrix and increased tumor cell mobility, which is necessary for metastasis. Furthermore, the role of EMT in drug resistance of CRC is documented [3, 4]. Therefore, therapeutic modalities that target EMT can be effective in efficient therapy of CRC [5].

Annexin A5 (*ANXA5*) is a cytosolic protein that belongs to the annexin family, multifunctional Ca2+ binding proteins that bind to membrane phospholipids [6]. ANXA5 also exists in the extracellular space, possibly due to unconventional secretory pathways [7]. ANXA5 plays important roles in several cellular activities including cell membrane repair, differentiation, and apoptosis [8]. The possible role of ANXA5 in various aspects of tumorigenesis was indicated by numerous in vivo and in vitro studies [6]. Sun et al. [9] showed that *ANXA5* overexpression in hepatocarcinoma cells enhanced proliferation and invasion of tumor cells. Similarly, in glioma cells, *ANXA5* upregulation promoted invasiveness of tumor cells [10]. In contrast, anti-tumor effects of *ANXA5* were revealed in some tumors such as melanoma [11], gastric cancer [12], non-Hodgkin’s lymphoma cell lines [13], and lung carcinoma [14], suggesting tumor-specific effects of *ANXA5*. Increased serum level of ANXA5 that was associated with increased tumor grade as well as lymph node metastasis have been shown in some patients with CRC [15]. However, to the best of our knowledge, the role of *ANXA5* in invasiveness of CRC cells has not been investigated. In the present study, we evaluated the effects of *ANXA5* overexpression on invasiveness of HCT 116 cells, a highly aggressive CRC cell line. Furthermore, the effects of *ANXA5* upregulation on the expression of major genes involved in EMT were evaluated.

## MATERIALS AND METHODS

**Cell culture: **The human colorectal cancer cell line HCT116 was purchased from Pasteur Institute (Iran) and cultured in complete RPMI 1640 supplemented with 10% of FBS (Gibco, USA) and 100 U/ml penicillin-streptomycin. Culture conditions were 5% CO_2_ and 37°C. 

**Construction of** **pCMV6-AC-IRES-GFP-ANX-A5 plasmid** **:** To overexpress *ANXA5*, *ANXA5* cDNA was cloned into a pCMV6-AC-IRES-GFP plasmid (Showed in supplementary data)(Origene Technologies Inc., USA) under expressional control of CMV promoter [16]. In brief, total RNA was extracted from HEK 293 cells, using RNX-Plus reagent (Sinaclon, Iran), according to the maufacturerer’s instruction. *ANXA5* cDNA was synthesized using 1.5 μg of total RNA and RevertAid First Strand cDNA Synthesis Kit (Thermo Fisher Scientific, USA). PCR amplification was performed using Taq DNA Polymerase Master Mix RED (Amplicon, Denmark) and *ANXA5* gene-specific primers (forward: 5’-CTT AGG ATC CAT GGC ACA GGT TCT CAG AGG-3’; reverse: 5’-CAG TTT CTA GAT TAG TCA TCT TCT CCA CAG AGC A-3’). The PCR reaction was performed by 95 ͦC first denaturation, followed by 35 cycles with 30 sec at 95 ͦC, 30 sec at 59 ͦC and 30 sec at 72 ͦC and ended by a final extension at 72 ͦC for 5 min. Both ANXA5 PCR product and pCMV6-AC-IRES-GFP were digested by BamH*I* and XbaI enzymes (Thermo Fisher Scientific, USA) to obtain the sticky ends. Then, they were ligated using T4 DNA ligase (Thermo Fisher Scientific, USA). The mock plasmid used in this study was pCMV6-AC-IRES-GFP plasmid that expressed *GFP* gene under the control of the CMV promoter. The mock and recombinant plasmids were transformed into DH5α competent cells using the calcium chloride (CaCl_2_) method. The cells were recovered in 1 ml Luria-Bertani (LB) broth and then incubated for 60 minutes at 37°C with shaking. The cells were cultured on LB-agar plates containing 100 μg/ml ampicillin and incubated at 37°C overnight to select the transformed colonies. The accuracy of cloning was verified using sequencing.

**HCT116 transfection with recombinant plasmid: **HCT116 cells were classified into three groups including un-transfected control cells (Control), mock plasmid- transfected cells (Mock), and cells transfected with pCMV6-AC-IRES-GFP-*ANXA5* plasmid (ANXA5). Recombinant plasmid was transfected into HCT116 cells using Lipofectmine 3000 (Invitrogen, USA), according to the manufacturer’s instruction. Fluorescent microscopy was applied to evaluate the efficiency of transfection (Cells with green fluorescence emission were considered as transfected cells). Transfection efficiency was evaluated using counting GFP expressing cells by fluorescent microscope 24, 48, and 72 h after transfection. In addition, RT-qPCR method using *ANXA5* specific primers (supplementary data) was applied to confirm the overexpression of *ANXA5*. In brief, 72 h after transfection, the cells were harvested and total RNA was isolated using Trizol reagent (BD Biosciences, USA). Total RNA was used to synthesis cDNA using a cDNA synthesis kit (Takara, Japan). Real-time PCR was conducted by Rotor gene 6000 (Qiagen, Germany) using SYBR green master mix in a total reaction volume of 20 µl. Thermal cycling was set-up as follows: 95ºC for 30s followed by 40 cycles of 95ºC for 30s, 58ºC for 30s and 72ºC for 30s. All reactions were repeated at least twice. The fold change in *ANXA5* mRNA expression was calculated using 2^-ΔΔCt^ formula. 

**MTT assay: **MTT (Dimethyl-thiazolyl diphenyl tetrazolium bromide) assay was used to evaluate the effects of ANXA5 overexpression on HCT116 cell viability. Briefly, 48 hours after the transfection, the cells were exposed to 5 mg/ml of MTT (Sigma Aldrich, USA) for four hours in 37°C. Afterwards, 100 μL of dimethyl sulfoxide (DMSO; Sigma Aldrich, USA) was added to each well and the plates were left in dark for 10 min. Finally, the absorbance of each well was measured in 570 nm wavelength, using an ELISA reader instrument.

**Transwell migration assay:** Transwell migration assay using polycarbonate inserts (Pore size: 8 μm; Corning®, USA) was applied to assess the effect of *ANXA5* overexpression on HCT116 cell migration [17]. In brief, 7×10^3^ HCT116 were diluted in 100 μl of serum-free media and seeded in the upper chamber of the polycarbonate inserts. The lower chambers were filled with 600 μl of the complete medium as the chemoattractant agent. After 8 hours, the cells under the lower surface of the insert were fixed by methanol and stained with 0.5% crystal violet both for 20 min. Finally, the migrated cells were counted under the inverted microscope with 400× magnification, in 5 random fields. 

**Wound healing assay: **To evaluate the effect of *ANXA5* overexpression on wound closure, the cultured cells were scratched by the micropipette tip and then pictured them at 0, 24, 48, and 72 hr after transfection. The area of wound closure was calculated using ImageJ software, and the percentage of wound closure was calculated. 

**Assessment of gene expression by RT-qPCR: **RT-qPCR was applied to evaluate the impact of *ANXA5 *overexpression on the expression of the genes involved in EMT. In brief, total RNA was isolated from the transfected and control cells by RNX-Plus reagent (Sinaclon, Iran), according to the manufacturer’s instruction. Then, cDNA was synthesized using 2 μg of RNA and RevertAid First Strand cDNA Synthesis Kit (Thermo Fisher Scientific, USA). RT-qPCR amplification was performed using RealQ Plus Master Mix Green (Ampliqon, Denmark) and Rotor-Gene 6000 (Qiagen, Germany) with specific primers (Supplementary data) [18]. Phosphoglycerate Kinase (*PGK*) was used as a reference gene.

**Statistical analysis: **SPSS version 15 was applied to analyze the data. Experiments were performed in at least three independent test. The data were analyzed using Kruskal-Wallis test and reported as mean ± SD. A p-value <0.05 was considered statistically significant. 

## RESULTS

**Figure 1:**
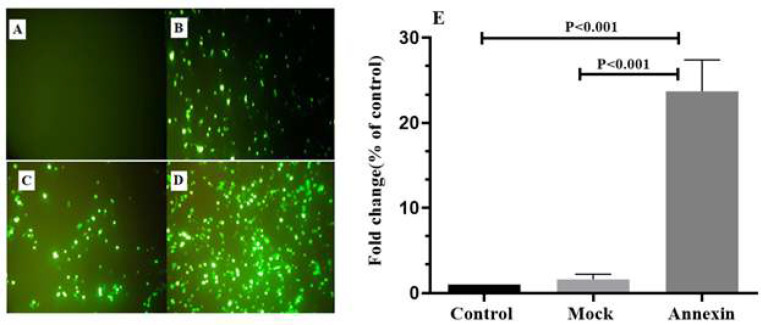
GFP expression was increased following transfection of HCT116 cells with mock and annexin-A5 harboring plasmids. A: control group, B: mock group, C: ANXA5 group 24 hours after transfection, D: ANXA5 group 48 hours after transfection. *ANXA5* was overexpressed following transfection of HCT116 cells with annexin-A5 harboring plasmids compared to control and mock group (E).

The HCT116 cells were transfected with a recombinant plasmid harboring *ANXA5* gene or mock plasmid using lipofectamine 3000. Counting the GFP expressing HCT116 cells using fluorescent microscopy revealed that the efficiency of transfection was at least 80% (Fig. 1). The results of RT-qPCR showed that *ANXA5* was overexpressed in ANXA5 group (23.7±3.7-fold) compared to the mock and control groups (Fig. 1).

**Figure 2:**
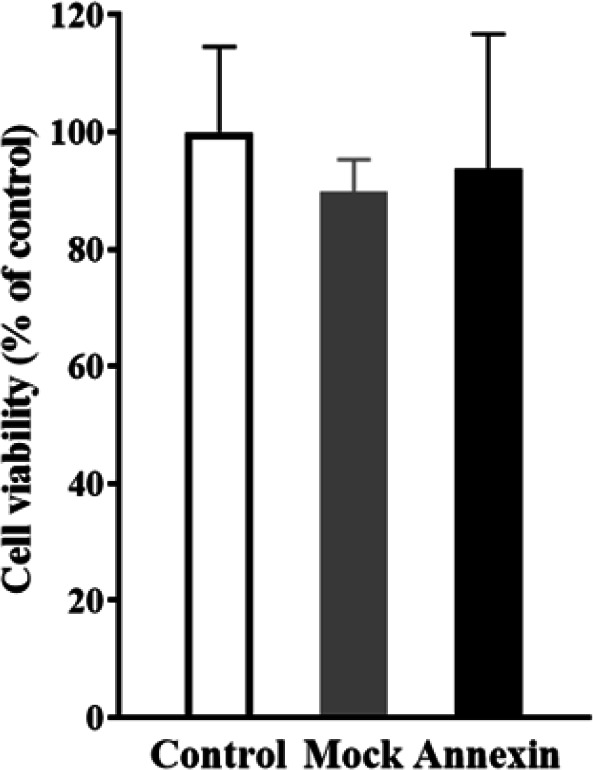
The effects of *ANXA5* overexpression on HCT116 cells viability. The data are mean ± SD of at least three independent experiments.

To evaluate the contribution of cell death in the observed effects of *ANXA5* overexpression, the viability of control, mock, and *ANXA5* overexpressed cells were measured using MTT assay. As shown in figure 2 , the overexpression of *ANXA5* had no significant effects on the cell viability of HCT116 cells compared to the mock and control groups. 

**Figure 3:**
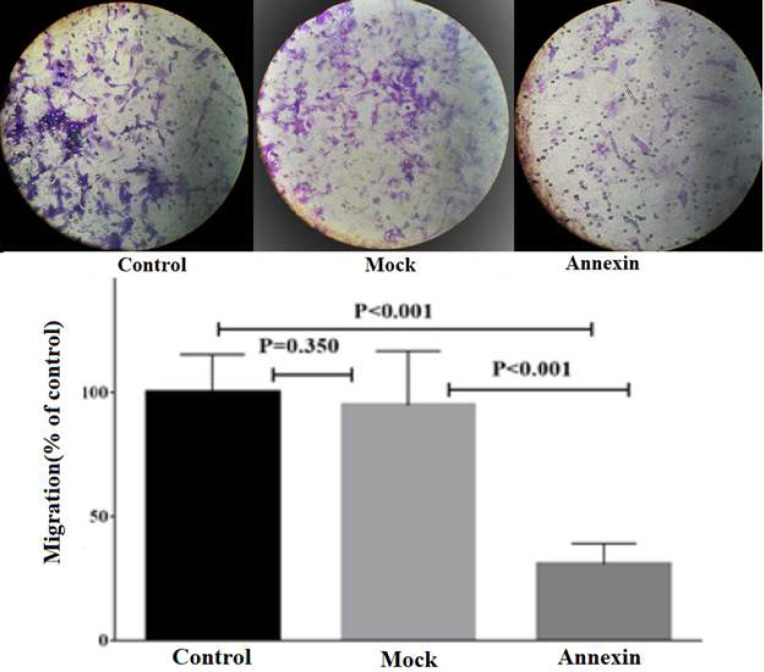
The effects of annexin-A5 overexpression on the number of migrated HCT116 cells in transwell migration assay. The data are mean ± SD of at least three independent experiments.

Transwell migration assay and wound healing method were used to investigate the effects of *ANXA5* overexpression on invasive abilities of HCT 116 cells. The number of migrated cells in *ANXA5*-overexpressing cells was significantly lowered compared to the mock-transfected and control cells; however, no significant difference was observed among the mock plasmid-transfected and control cells, suggesting inhibitory effects of *ANXA5* on cell migration (Fig. 3). 

**Figure 4:**
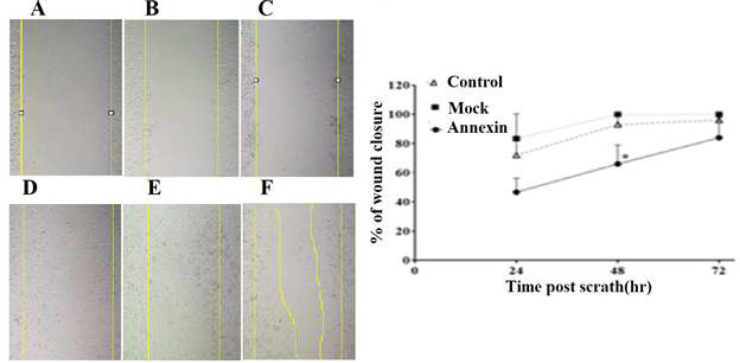
The effects of annexin-A5 overexpression on the migration of HCT 116 cells in wound healing assay. A, B, and C illustrate wound area immediately after scratching in the control, mock, and Annexin-A5 groups, respectively. D, E, and F show wound area 48 hours after scratching in the control, mock, and ANXA5 groups, respectively. The graph illustrates the percentage of wound closure 24, 48, and 72 hours after scratching. *Shows a significant difference between ANXA5 group compared to the mock (p=0.022) and control cells (p=0.045).

Figure 4 shows the effect of *ANXA5* overexpression on HTC 116 cell motility in wound healing assay. The wound closure area was measured at 0, 24, 48, and 72 hours after scratching the monolayer cells. The percentage of wound closure was significantly lowered in ANXA5-transfected cells compared to the mock and control group 48 hours after scratching. However, no significant difference was observed between the mock and control group at 24, 48, and 72 hours after scratching (Fig. 4). These results strongly support the negative impact of *ANXA5* overexpression on the migration of HCT 116 cells. 

**Figure 5:**
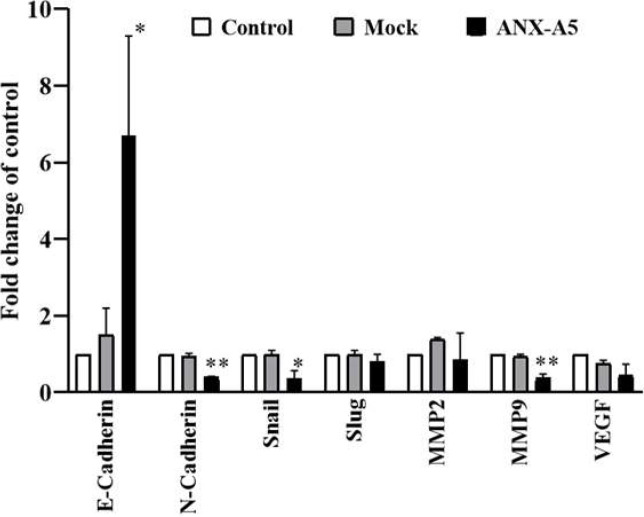
The represented data are mean ± SD. *ANXA5*: Annexin plasmid-transfected cells; Mock: Mock plasmid-transfected cells; Control: non-transfected cells. MMP: matrix metalloproteinase; VEGF: vascular endothelial growth factor. *p<0.05, **p<0.01 compared to Mock group.

Figure 5 compares the expression of genes involved in EMT between *ANXA5*-transfected, mock-transfected, and un-transfected HCT116 cells. A significant increase in the expression of E-cadherin was observed in *ANXA5*-transfected compared to mock-transfected and un-transfected HCT116 cells (p=0.017). Furthermore, the mRNA levels of N-cadherin, Snail, and *MMP-9* were significantly decreased in the *ANXA5*-overexpressed cells compared to mock-transfected and non-transfected control cells. The expression of *MMP-2* and *VEGF* was not significantly altered following *ANXA5* overexpression (Fig. 5).

## DISCUSSION

Metastasis is the major cause of CRC-related death; therefore, many efforts are being made to find a promising approach that can target CRC metastasis. Analyzing the results of transwell migration and wound healing assay in the present study revealed that *ANXA5* overexpression could inhibit migration of HCT 116 cells, a highly invasive CRC cell line. Our data also revealed that *ANXA5* overexpression reduces the expression of EMT-related genes, suggesting possible beneficial effects of *ANXA5* in inhibiting metastasis and expansion of CRC tumor cells.

The findings of MTT assay showed that *ANXA5* overexpression had no significant effects on viability of HCT116 cells. However, the data of wound healing and transwell migration assay evidently revealed that *ANXA5 *overexpression could decrease the motility and migration of HCT 116 cells. These results are in line with those of a previous study in cervical cancer lines [19], where *ANXA5* overexpression decreased migration and invasion of Hela and SiHA cells. Similarly, Balch et al. [3] treated Lewis lung carcinoma cells with ANXA5 protein extracellularly and observed a significant suppression of cell migration, apparently through binding to cell membrane acidic phospholipids and inhibiting formation of cell membrane protrusions, which are necessary for cell motility. Furthermore, it has been demonstrated that stable overexpression of *ANXA5* in non-Hodgkin’s lymphoma cell lines could inhibit cell invasion through decreasing MMP-9 expression and ANXA5 knockdown using specific shRNA increased MMP-9 activity and tumor cell invasion; this supports the possible role of ANXA5 in inhibiting invasiveness characteristics of these tumor cells [13]. Conversely, in some tumor cells such as oral carcinoma cells [20] and murine hepatocarcinoma cells [21], as well as several normal cells including human keratinocytes [22] and corneal epithelium [23], ANXA5 upregulation has increased cell migration, suggesting diverse effects of ANXA5 in different cell types. These contradictory findings can be related to use of different methods for treating with ANXA5, i.e., extracellularly with ANXA5 protein or intracellularly through gene overexpression, applying different strategies for the cell migration assay, and the type of studied cells, normal vs. tumorous. 

Due to the crucial role of EMT in the CRC metastasis, we determined whether *ANXA5* overexpression could alter the expression of key molecules involved in this phenomenon including E-cadherin, N-cadherin, and MMPs. As expected, analysis of the real-time PCR data revealed a substantial increase in E-cadherin mRNA (6.7-fold) and a significant decrease in N-cadherin mANA (0.4-fold) following *ANXA5* overexpression; this suggests the possible role of ANXA5 in –“cadherin switching”, a well-established event in the EMT processing [24]. Similarly, Gong et al. [14] showed an abundant increase in E-cadherin expression following overexpression of *ANXA5* in the lung squamous carcinoma cell lines. It has been reported that increased expression of Snail and Slug transcription factors plays a crucial role in the control of E-cadherin gene expression [18]. These transcription factors repress E-cadherin expression through binding to E-box elements in the promoter of the E-cadherin gene [25]. Furthermore, it was shown that Snail overexpression repressed expression of E-cadherin and induced EMT phenotype in HCT116 cells [26]. Findings of the present study revealed that *ANXA5* overexpression reduces Slug and Snail expression, suggesting a possible role of decreasing expression of these transcription factors in the ANXA5-induced E-cadherin expression. 

The critical role of MMP-9 in EMT processing and tumor cell invasion is well documented [27]. In patients with CRC, the association of increased expression and plasma level of MMP-9 with lymph node invasion, poor prognosis, and worse survival has been reported in several studies [27-29]. Analysis of the real-time PCR data showed that *ANXA5* overexpression could decrease the MMP-9 expression, indicating that the decrease in the expression of MMPs might be involved in the observed anti-invasive effects of *ANXA5*. In accordance with our data, suppressing effects of ANXA5 on MMP-9 expression has been revealed in cervical cancer lines [19].

Contrary to findings of this study, Sun et al [15] measured the serum level and expression of *ANXA5* in tumor tissues of 93 patients with CRC and 40 healthy individuals and showed a significant higher level of *ANXA5* in the serum of CRC patients compared to control group. They also detected a significant higher tissue expression of *ANXA5* in CRC patients with lymph node metastasis, which was positively correlated with the stage of CRC and serum level of *ANXA5*. The authors concluded that *ANXA5* expression in CRC tissues is related to lymph node metastasis and tumor grade. Therefore, further investigations are recommended to confirm the results of our study.

This study had several limitations. First of all, we evaluated the effects of *ANXA5* overexpression on viability and invasiveness of HCT116 cells. Although this cell line is known as a highly invasive CRC cells and was a good candidate for our study; however, it is necessary to evaluate the effects of *ANXA5* on the other CRC cell lines. Furthermore, the expression of *ANXA5 *was investigated at level of mRNA; further studies using a specific antibody against *ANXA5* is warranted to evaluate the expression of this gene at the protein level as well as subcellular localization. 

 All in all, our data revealed that *ANXA5* overexpression could inhibit the migration of HCT116 cells. Furthermore, *ANXA5 *upregulation altered the expression of the genes involved in EMT, including E-cadherin, N-cadherin, Snail, Slug, and MMP-9; this shows potential beneficial effects of *ANXA5* in preventing CRC metastasis. However, additional investigations are warranted to shed light on the signaling pathways affected by *ANXA5* in CRC cells. 

## Conflict of Interest:

 The authors disclose no conflicts of interest.
